# Safety and efficacy of sintilimab versus pembrolizumab in the treatment of advanced or recurrent pediatric malignancies: a real-world study in China

**DOI:** 10.3389/fimmu.2025.1608844

**Published:** 2025-06-06

**Authors:** Lei Mao, Mengzhen Li, Linnan Wu, Juan Wang, Yi Que, Feifei Sun, Junting Huang, Suying Lu, Zijun Zhen, Jia Zhu, Mengjia Song, Yizhuo Zhang

**Affiliations:** Department of Pediatric Oncology, Sun Yat-sen University Cancer Center, State Key Laboratory of Oncology in South China, Collaborative Innovation Center for Cancer Medicine, Guangzhou, China

**Keywords:** pediatric malignancies, PD-1 inhibitors, sintilimab, pembrolizumab, safety, efficacy

## Abstract

**Background:**

Programmed death receptor 1 (PD-1) inhibitors have shown durable response and mild adverse events in adult malignancies. However, study on PD-1 inhibitors in pediatric patients remains limited, and a direct comparison of distinct PD-1 inhibitors in pediatric tumors is lacking.

**Methods:**

We conducted a retrospective analysis of 75 pediatric patients with advanced or recurrent malignancies treated with either Sintilimab-based (n=53) or Pembrolizumab-based (n=22) regimens. The primary endpoints included treatment-related adverse events (TRAEs) and objective response rate (ORR), and the second endpoints included progression-free survival (PFS) and overall survival (OS).

**Results:**

The incidence of hypothyroidism, hyperthyroidism, pneumonia, increased ALT/AST, gastroenteritis, and rash following immune checkpoint inhibitor therapy showed no significant differences between the Sintilimab group and the Pembrolizumab group (all P>0.05). Cardiovascular Adverse Events (CVAEs) occurred in 26.0% (15/53) of Sintilimab-treated patients versus 40.0% (8/20) of Pembrolizumab-treated patients (P=0.26). In the lymphoma cohort (n=13), 88.9% of Sintilimab-treated patients and 75.0% of Pembrolizumab-treated patients achieved complete response (CR) or partial response (PR) (P=0.54). The median PFS and OS were not reached in either group. In the non-lymphoma cohort (n=53), 40.5% of Sintilimab-treated patients and 25.0% of Pembrolizumab-treated patients achieved CR or PR (P=0.18). Among 39 patients who had received ≤ 2 prior treatment lines, the PFS and OS showed no significant differences between the Sintilimab (n=30) and Pembrolizumab (n=9) groups (P=0.28 and P=0.09, respectively). Similarly, among 14 patients who had received>2 prior treatment lines, no significant differences in PFS and OS were observed between the Sintilimab(n=7) and Pembrolizumab(n=7) groups (P=0.33 and P=0.15, respectively).

**Conclusions:**

Sintilimab demonstrated favorable tolerability and efficacy in pediatric patients with malignancies, with a safety and efficacy profile comparable to Pembrolizumab. For pediatric patients with advanced or recurrent malignancies receiving immune checkpoint inhibitor therapy, long-term monitoring of thyroid and cardiac function is recommended.

## Introduction

Through comprehensive treatment modalities, including nonspecific cytotoxic chemotherapy, surgery, radiotherapy, and targeted therapy, the 5-year survival rate of pediatric patients with malignancies has significantly improved, reaching 85–86% (1, 2). Unfortunately, the prognosis for children with advanced or recurrent malignancies remains poor, with a 10-year overall survival (OS) and progression free survival (PFS) of only 24.5% and 18.4%, respectively (3, 4). There is an urgent need for safe and effective novel therapeutic strategies to improve outcomes for pediatric patients with advanced or recurrent malignancies.

Over the past decade, programmed cell death-1 (PD-1) inhibitors have demonstrated durable responses and mild adverse events (AEs) in adult patients with advanced or recurrent cancers across numerous clinical trials. However, study regarding the safety and efficacy of PD-1 inhibitors in pediatric patients is limited (2, 4). Results from an interim analysis of the KEYNOTE-051 trial indicated that Pembrolizumab exhibited anti-tumor activity in pediatric patients with advanced melanoma and PD-L1-positive, advanced, relapsed, or refractory solid tumor or lymphoma (5). Sintilimab, a fully humanized immunoglobulin G4 anti-PD-1 monoclonal antibody, has demonstrated promising tolerability and anti-tumor activity in pediatric patients with advanced or recurrent malignancies in a phase I study (NCT04400851) (6). Previous studies have shown that PD-1 inhibitors are more effective in pediatric patients with lymphoma than other malignant solid tumors (7, 8). Notably, pharmacodynamic study has revealed that Sintilimab binds to human PD-1 with greater affinity, engages more PD-1 molecules on CD3^+^ T-cells, and exhibits superior T-cell activating characteristics (9). These findings highlight the potential of Sintilimab as a promising therapeutic option for pediatric malignancies.

To date, no direct comparisons of Sintilimab and Pembrolizumab in pediatric patients have been conducted. Given the limited data, we performed a retrospective study to compare the safety and efficacy of Sintilimab and Pembrolizumab, both in combination with chemotherapy, radiotherapy, or targeted therapy, in pediatric patients with advanced or recurrent malignancies in a real-world setting. Subgroup analyses were performed based on tumor type and treatment line.

## Patients and methods

### Patients

This retrospective study was performed at Sun Yat-sen University Cancer Center evaluating pediatric patients with advanced or recurrent malignancies who received Sintilimab-based or Pembrolizumab-based regimens outside of clinical trials between April 2017 and August 2024.

The inclusion criteria were as follows:1) histologically or cytologically confirmed advanced malignancies or had clear evidence of recurrence; 2) age at initiation of Sintilimab or Pembrolizumab <18 years; 3) receiving at least one cycle of Sintilimab-based or Pembrolizumab-based regimens and at least one follow-up visit; 4) having measurable lesions. The exclusion criteria were as follows:1) patients who switched to other kinds of PD-1 or PD-L1 inhibitor, such as Atezolizumab, Nivolumab, Toripalimab, Camrelizumab, Tislelizumab; 2) Patients who received a PD-1 antibody as part of a clinical trial.

Subgroup analyses were performed based on tumor type, categorizing patients into lymphoma and non-lymphoma groups. The study arms were defined as follows: Arm A included pediatric patients diagnosed with advanced or recurrent lymphoma who received Sintilimab; Arm B included pediatric patients diagnosed with advanced or recurrent non-lymphoma malignancies who received Sintilimab; Arm C included pediatric patients diagnosed with advanced or recurrent lymphoma who received Pembrolizumab; Arm D included pediatric patients diagnosed with advanced or recurrent non-lymphoma malignancies who received Pembrolizumab. Subgroup analyses were also performed based on treatment line among patients with non-lymphoma group.

This study was approved by the Institutional Review Board and Ethics Committee of the Sun Yat-sen University Cancer Centre (B2024-681-01) and was conducted following the Code of Ethics of the World Medical Association (Declaration of Helsinki) for experiments involving humans and Good Clinical Practice. The requirement for written informed consent was waived.

### Data collection and outcome measurement

The demographic, tumor histology, age at initiation of Sintilimab or Pembrolizumab, treatment history, clinical efficacy, and toxicity for each patient were obtained from retrospective electronic medical records by investigators. PD-1 inhibitor dosing was performed in accordance with the manufacturer’s instructions: Pembrolizumab (2 mg/kg with a maximal dose of 200 mg, q3w), and Sintilimab(3mg/kg with a maximal dose of 200 mg, q3w).

The primary objective of this study was to describe treatment-related adverse events (TRAEs) and objective response rate (ORR), and the secondary objective was to describe progression-free survival (PFS) and overall survival (OS). ORR is the proportion of patients who achieved complete response (CR) or partial response (PR). The disease control rate (DCR) is the proportion of patients who achieved CR, PR or SD. PFS was calculated as the date between the initiation of Sintilimab or Pembrolizumab and disease progression or death from any cause. OS was defined as the date between the initiation of Sintilimab or Pembrolizumab and death from any cause, or lost to follow-up. The Response Evaluation Criteria in Solid Tumors version 1.1 and Lugano classification were used to evaluate the response of solid tumors and lymphoma, respectively. Toxicity was evaluated according to the National Cancer Institute’s Common Terminology Criteria for Adverse Events (CTCAE), version 5.0. Both PFS and OS were estimated utilizing the Kaplan–Meier statistical method.

### Statistical analysis

All statistical analyses were performed using SPSS software (ver. 21, IBM Inc, IL, USA) and GraphPad Prism (ver. 8.0.0 for Windows, GraphPad Software, San Diego, California USA). A two-sided P-value <0.05 was considered significant for all analysis. The ORR, DCR, PFS, and OS were evaluated in the full analysis set (FAS), defined as all patients who met the study inclusion criteria received at least one dose of Sintilimab or Pembrolizumab, and were followed up at least once. Safety and tolerability were evaluated in the safety set (SS), defined as all patients who received at least one dose of Sintilimab or Pembrolizumab, and had at least one post-baseline safety evaluation.

## Results

### Patients’ characteristics

**Figure 1:**
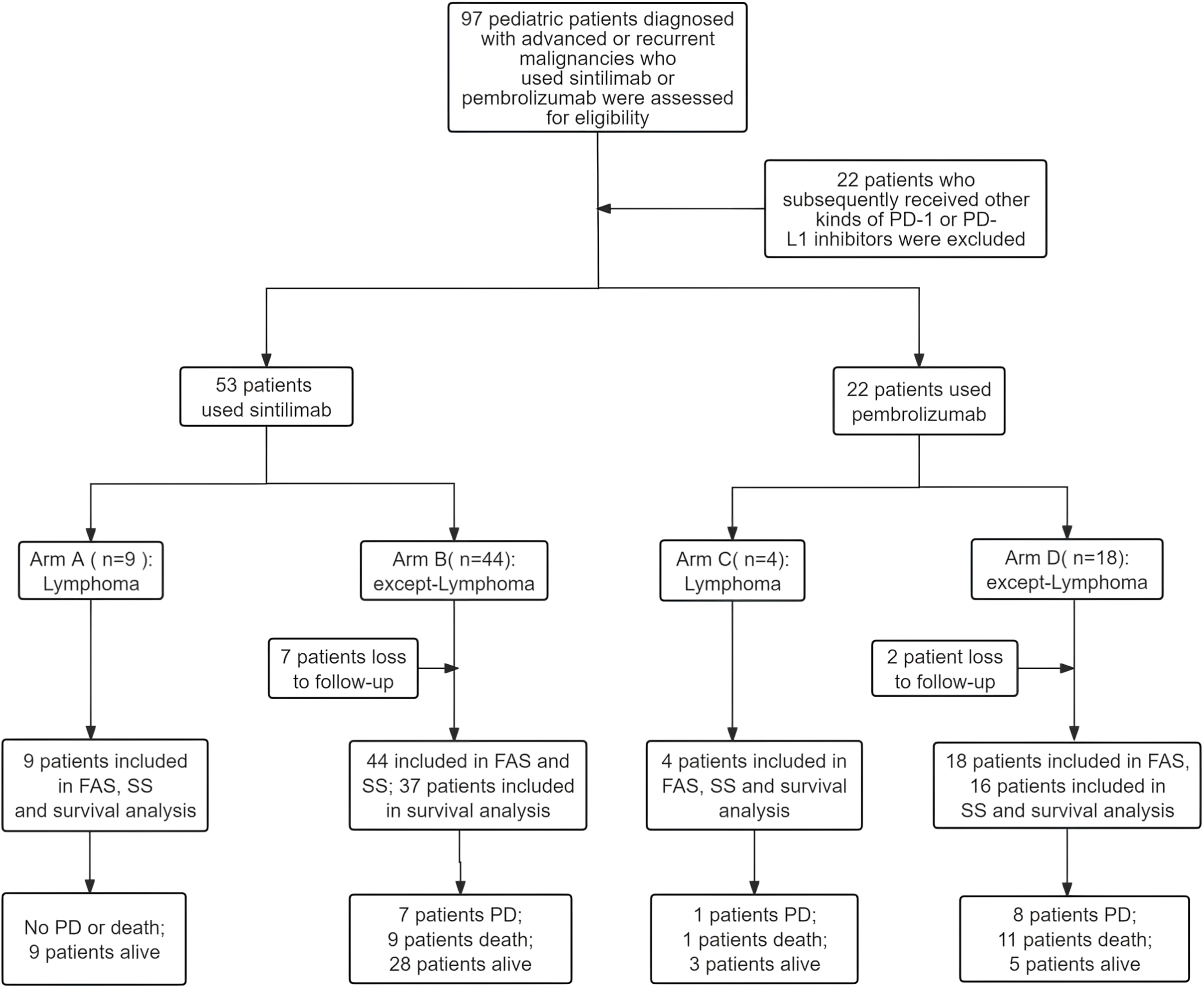
Flow chart of patient screening. PD-1, Programmed cell death receptor-1; PD-L1, Programmed death ligand-1; PD, Progressive disease. FAS: Patients who met the study inclusion criteria received at least one dose of the drug and were followed at least once. SS: Patients who received at least one dose of the drug and had at least one post-baseline safety evaluation.

From April 2017 to August 2024, a total of 97 pediatric patients diagnosed with advanced or recurrent malignancies who received Sintilimab or Pembrolizumab outside of clinical trials were assessed for eligibility. 22 patients who subsequently received other kinds of PD-1 or PD-L1 inhibitors were excluded. 53 patients who received Sintilimab and 22 patients who received Pembrolizumab were eligible for efficacy evaluation and safety analysis (**Figure 1**).

The baseline characteristics of patients are listed in **Table 1**. A total of 75 patients treated with Sintilimab (n=53) and Pembrolizumab (n=22) were eligible for safety and efficacy assessments, including lymphoma (13/75, 17.3%), bone and soft tissue sarcoma (26/75, 34.7%), central nervous system tumor (6/75, 8.0%), and the other solid tumor (30/75, 40.0%). For patients with lymphoma, the median ages were 7.0 years (range: 4-17) for those receiving Sintilimab (Arm A, n=9) versus 13.5 years (range: 6-15) for Pembrolizumab (Arm C, n=4), with a P value of 0.45. Among patients with non-lymphoma malignancies, the median ages were 8.5 years (range: 1-16) for Sintilimab (Arm B, n=44) and 7.5 years (range: 3-17) for Pembrolizumab (Arm D, n=18), with a P value of 0.99. The median number of prior treatment lines before the PD-1 inhibitors was 1 (range 1-5), and a total of 18 patients received > 2 prior treatment lines. The median number of treatment cycles with PD-1 inhibitors was 5 (range 1-30). The treatment regimens included PD-1 inhibitor combined chemotherapy (52/75, 69.3%), combined radiotherapy (38/75, 50.7%), and combined targeted therapy (38/75, 50.7%). The treatment regimen showed no significant difference neither in Arm A and Arm B, nor in Arm C and Arm D.

**Table 1:** Baseline characteristics of patients.

Characteristics	Arm A (n=9)	Arm C (n=4)	P value	Arm B (n=44)	Arm D (n=18)	P value
Gender, Male, n (%)	8 (88.9%)	4 (100%)	0.51	24 (54.6%)	9 (50.0%)	0.75
Age (years), median (range)	7 (4–17)	13.5 (6–15)	0.45	8.5 (1–16)	7.5 (3–17)	0.99
Tumor type						
Classical Hodgkin Lymphoma	4 (44.5%)	0	0.12	0	0	–
Primary Mediastinal Large B-Cell Lymphoma	1 (11.1%)	2 (50.0%)	0.14	0	0	–
NK/T-cell lymphoma	4 (44.5%)	2 (50.0%)	0.86	0	0	–
Central nervous system malignancies	0	0	–	6 (13.6%)	0	0.1
Neuroblastoma	0	0	–	5 (11.4%)	4 (22.2%)	0.48
Melanoma	0	0	–	3 (6.8%)	3 (16.7%)	0.24
Nasopharyngeal carcinoma	0	0	–	2 (4.6%)	0	0.36
Lymphoepithelioid carcinoma	0	0	–	2 (4.6%)	0	0.36
Adrenocortical carcinoma	0	0	–	1 (2.3%)	3 (16.7%)	0.05
Rhabdomyosarcoma	0	0	–	6 (13.6%)	1 (5.6%)	0.33
Ewings sarcoma	0	0	–	4 (9.1%)	1 (5.6%)	0.63
Alveolar soft-part sarcoma	0	0	–	2 (4.6%)	2 (11.2%)	0.7
Malignant Rhabdoid tumor	0	0	–	3 (6.8%)	1 (5.6%)	0.86
Undifferentiated sarcoma	0	0	–	0	1 (5.6%)	0.12
Hepatoblastoma	0	0	–	0	1 (5.6%)	0.12
Desmoplastic small round cell tumor	0	0	–	1 (2.3%)	1 (5.6%)	0.51
Germinoma	0	0	–	3 (6.8%)	0	0.26
Hepatocellular Carcinoma	0	0	–	1 (2.3%)	0	0.52
Renal Cell Carcinoma	0	0	–	2 (4.6%)	0	0.36
Chordoma	0	0	–	1 (2.3%)	0	0.52
Synovial sarcoma	0	0	–	1 (2.3%)	0	0.52
Inflammatory myofibroblast tumor	0	0	–	1 (2.3%)	0	0.52
Median number of prior treatment line (range)	1 (1–3)	2 (1–5)	0.16	1 (1–5)	2 (1–4)	0.27
Number of treatment line>2, n (%)	1 (11.1%)	2 (50.0%)	0.20	8 (18.2%)	7 (38.9%)	0.16
Median cycle of PD-1 inhibitor treatment (range)	7 (3–30)	6 (1–8)	0.26	4 (1–30)	4 (1–14)	0.62
Chemotherapy, n (%)	8 (88.9%)	3 (75.0%)	0.54	32 (72.7%)	9 (50.0%)	0.09
Radiotherapy, n (%)	4 (44.5%)	1 (25.0%)	0.52	23 (52.3%)	10 (55.6%)	0.81
Targeted drug therapy, n (%)	4 (44.5%)	0	0.12	23 (52.3%)	11 (61.1%)	0.53

Arm A: Pediatric patients diagnosed with advanced or recurrent lymphoma who received Sintilimab. Arm B: Pediatric patients diagnosed with advanced or recurrent non-lymphoma malignancies who received Sintilimab. Arm C: Pediatric patients diagnosed with advanced or recurrent lymphoma who received Pembrolizumab. Arm D: Pediatric patients diagnosed with advanced or recurrent non-lymphoma malignancies who received Pembrolizumab.

### Treatment-related toxicity

A total of 73 patients were included in the safety evaluation. Treatment-related adverse effects (TRAEs) following PD-1 inhibitors were shown in **Table 2**. The incidence of hypothyroidism, hyperthyroidism, and pneumonia were slightly higher in the Sintilimab group compared with the Pembrolizumab group, with the incidence of 11.3% (6/53) versus 10.0% (2/20), 17.0% (9/53) versus 15.0% (3/20), and 34.0% (18/53) versus 15.0% (3/20), respectively. The incidence of cardiovascular adverse events (CVAEs), defined as myocarditis, pericarditis, new advanced conduction disease (second- or third- degree heart block), new early conduction abnormality on electrocardiogram (ECG), new asymptomatic rise in Brain Natriuretic Peptide (BNP) or N-Terminal pro BNP, new onset Left Ventricle Systolic Dysfunction, between the Sintilimab group compared with the Pembrolizumab group was 26.0% (15/53) versus 40.0% (8/20) (P=0.26). The incidence of increased ALT/AST, gastroenteritis, and rash between the Sintilimab group compared with the Pembrolizumab group were 28.3% (15/53) versus 40.0% (8/20), 3.8% (2/53) versus 15.0% (3/20), 3.8% (2/53) versus 5.0% (1/20), respectively. All adverse reactions had no significant differences between the Sintilimab group and the Pembrolizumab group. No autoimmune diabetes mellitus and severe infusion reactions were observed in either Sintilimab or Pembrolizumab group.

**Table 2:** Treatment-related adverse effects following PD-1 inhibitors between Sintilimab group (N=53) and Pembrolizumab group (N=20).

Adverse events (Grade 1–2)*	Sintilimab group N (%)	Pembrolizumab group N (%)	P value
Infusion reactions	0	0	-
Hypothyroidism	6 (11.3%)	2 (10.0%)	0.9
Hyperthyroidism	9 (17.0%)	3 (15.0%)	1.0
Myositis	1 (1.9%)	0	-
Autoimmune Diabetes Mellitus	0	0	-
Pneumonia	18 (34.0%)	3 (15.0%)	0.11
Increased ALT/AST	15 (28.3%)	8 (40.0%)	0.34
Gastroenteritis	2 (3.8%)	3 (15.0%)	0.12
Rash	2 (3.8%)	1 (5.0%)	1.00
CVAEs	14 (26.0%)	8 (40.0%)	0.26

*No Grade 3 or higher non-hematologic TRAEs were observed. ALT, Glutamic-pyruvic transaminase; AST, Glutamic oxalacetic transaminase; CVAEs, Cardiovascular Adverse Events.

Among the whole cohort patients (**Table 3**), 39.7% (29/73) experienced Grade 3–4 hematological TRAEs, mainly including granulocytopenia and anemia. No Grade 3 or higher non-hematological TRAEs were observed. The most common grade 1/2 non-hematologic TRAEs included anorexia (29/73, 39.7%), increased ALT/AST levels (23/73, 31.5%), pneumonia (21/73, 28.8%), fever (20/73, 27.4%), and fatigue (20/73, 27.4%). The incidence of decreased white blood cell count, fever, anorexia, thyroid dysfunction, pneumonia, and abdomen pain was higher in the Sintilimab group compared to the Pembrolizumab group, with all P values > 0.05. Conversely, the incidence of anemia, decreased neutrophils count, decreased platelets, fatigue, nausea, increased ALT/AST, pleural effusion, gastroenteritis, and rash was higher in the Pembrolizumab group compared to the Sintilimab group. Safety profiles were similar between patients with lymphoma and non-lymphoma malignancies in the Sintilimab and Pembrolizumab groups, with all P values > 0.05.

**Table 3:** Treatment-related adverse effects of Arm A (n=9) versus Arm C (n=4), Arm B (n=44) versus Arm D (n=16).

Adverse event	All, n (%)			Grade 3-5, n (%)			All, n (%)			Grade 3-5, n (%)		
	Arm A	Arm C	P value	Arm A	Arm C	P value	Arm B	Arm D	P value	Arm B	Arm D	P value
Anemia	8 (88.9%)	4 (100.0%)	0.51	7 (77.8%)	1 (25.0%)	0.08	33 (75.0%)	13 (81.3%)	0.87	15 (34.1%)	5 (31.3%)	0.84
Decreased white blood cell count	8 (88.9%)	4 (100.0%)	0.51	5 (55.6%)	2 (50.0%)	0.86	29 (65.9%)	8 (50.0%)	0.26	15 (34.1%)	4 (25.0%)	0.50
Decreased neutrophils count	8 (88.9%)	4 (100.0%)	0.51	6 (66.7%)	3 (75.0%)	0.77	24 (54.6%)	8 (50.0%)	0.76	15 (34.1%)	5 (31.3%)	0.84
Decreased platelets	5 (55.6%)	2 (50.0%)	0.86	2 (22.2%)	1 (25.0%)	0.92	21 (47.7%)	8 (50.0%)	0.88	9 (20.5%)	3 (18.8%)	0.88
Fever	4 (44.5%)	1 (25.0%)	0.52	-	-	-	14 (31.8%)	1 (6.3%)	0.09	-	-	-
Fatigue	2 (22.2%)	2 (50.0%)	0.34	-	-	-	11 (25.0%)	5 (31.3%)	0.88	-	-	-
Anorexia	3 (33.3%)	1 (25.0%)	0.77	-	-	-	20 (45.5%)	5 (31.3%)	0.32	-	-	-
Nausea	1 (11.1%)	1 (25.0%)	0.54	-	-	-	3 (6.8%)	3 (18.8%)	0.39	-	-	-
Thyroid dysfunction	5 (55.6%)	1 (25.0%)	0.52	-	-	-	10 (22.7%)	6 (37.5%)	1.0	-	-	-
Increased ALT/AST	3 (33.3%)	1 (25.0%)	0.77	-	-	-	10 (22.7%)	7 (43.8%)	0.23	-	-	-
Increased creatinine	0	0	-	-	-	-	3 (6.8%)	1 (6.3%)	0.94	-	-	-
Pneumonia	3 (33.3%)	2 (50.0%)	0.14	-	-	-	15 (34.1%)	1 (6.3%)	0.07	-	-	-
Pleural effusion	0	1 (25.0%)	0.13	-	-	-	1 (2.3%)	1 (6.3%)	0.48	-	-	-
Gastroenteritis	0	0	-	-	-	-	2 (4.6%)	3 (18.8%)	0.1	-	-	-
Abdomen pain	0	0	-	-	-	-	6 (13.6%)	2 (12.5%)	0.91	-	-	-
Rash	1 (11.1%)	0	0.33	-	-	-	1 (2.0%)	1 (6.3%)	0.22	-	-	-
Hypertension	0	0	-	-	-	-	2 (4.6%)	0	0.26	-	-	-
CVAEs	2 (22.2%)	1 (25.0%)	0.13	-	-	-	12 (27.0%)	7 (44.0%)	0.31	-	-	-

Arm A: Pediatric patients diagnosed with advanced or recurrent lymphoma who received Sintilimab. Arm B: Pediatric patients diagnosed with advanced or recurrent non-lymphoma malignancies who received Sintilimab. Arm C: Pediatric patients diagnosed with advanced or recurrent lymphoma who received Pembrolizumab. Arm D: Pediatric patients diagnosed with advanced or recurrent non-lymphoma malignancies who received Pembrolizumab. ALT, Glutamic-pyruvic transaminase; AST, Glutamic oxalacetic transaminase; CVAEs, Cardiovascular Adverse Events.

### Treatment responses

The efficacy data for the 66 patients in the full analysis set are summarized (**Table 4**). In the cohort of lymphoma patients, 88.9% (8/9) receiving Sintilimab in Arm A and 75.0% (3/4) receiving Pembrolizumab in Arm C achieved CR or PR (P =0.54). For those with non-lymphoma malignancies, 40.5% (15/37) in Arm B who received Sintilimab and 25.0% (4/16) in Arm D who received Pembrolizumab experienced similar responses (P=0.18). Disease progression occurred in 18.9% (7/37) of patients in Arm B, 25.0% (1/4) in Arm C, and notably, 50.0% (8/16) in Arm D. No statistically significant differences in ORR were observed between Arm A and Arm C, or between Arm B and Arm D.

**Table 4:** Tumor response in full analysis set.

Best overall response, n (%)	Arm A (n=9)	Arm C (n=4)	P value	Arm B (n=37)	Arm D (n=16)	P value	Arm A+B (n=46)	Arm C+D (n=20)	P value
Complete remission	5 (55.6%)	3 (75.0%)	0.52	6 (16.2%)	2 (12.5%)	0.73	11 (23.9%)	5 (25.0%)	0.93
Partial response	3 (33.3%)	0	0.21	9 (24.3%)	2 (12.5%)	0.55	12 (26.1%)	2 (10.0%)	0.25
Stable disease	1 (11.1%)	0	0.51	15 (40.5%)	4 (25.0%)	0.28	16 (34.8%)	4 (20.0%)	0.23
Progressive disease	0	1 (25.0%)	0.13	7 (18.9%)	8 (50.0%)	0.10	7 (15.2%)	9 (45.0%)	0.02
Objective response rate, %	8 (88.9%)	3 (75.0%)	0.54	15 (40.5%)	4 (25.0%)	0.18	23 (50.0%)	7 (35.0%)	0.26
Disease control rate, %	9 (100.0%)	3 (75.0%)	0.13	30 (81.1%)	8 (50.0%)	0.05	39 (84.8%)	11 (55.0%)	0.02

Arm A: Pediatric patients diagnosed with advanced or recurrent lymphoma who received Sintilimab. Arm B: Pediatric patients diagnosed with advanced or recurrent non-lymphoma malignancies who received Sintilimab. Arm C: Pediatric patients diagnosed with advanced or recurrent lymphoma who received Pembrolizumab. Arm D: Pediatric patients diagnosed with advanced or recurrent non-lymphoma malignancies who received Pembrolizumab.

### Progression-free survival and overall survival

**Figure 2:**
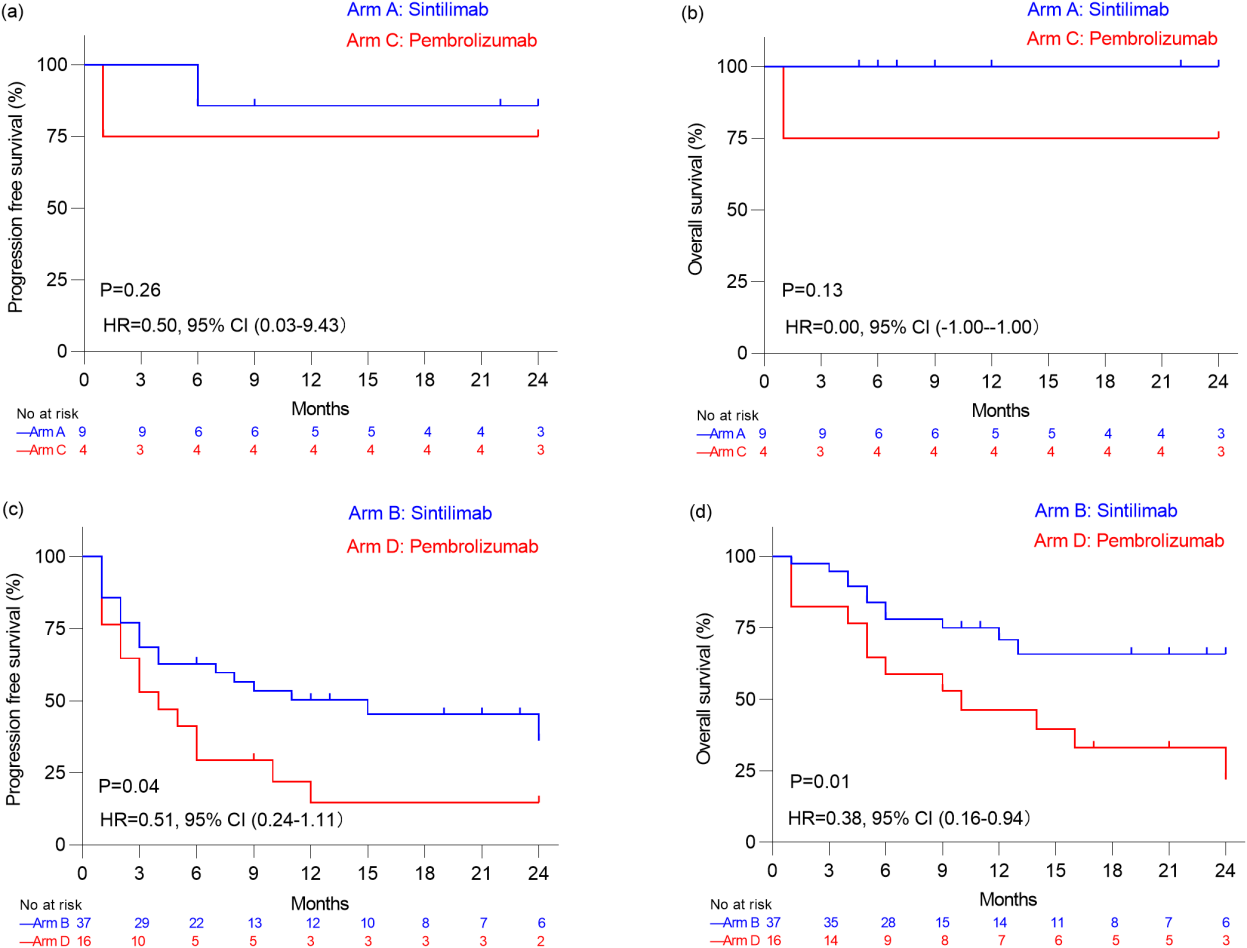
Kaplan–Meier analysis of progression free survival estimates **(a)** and overall survival estimates **(b)** in patients with advanced or recurrent Lymphoma who received Sintilimab (Arm A) and who received Pembrolizumab (Arm C). Kaplan–Meier analysis of progression free survival estimates **(c)** and overall survival estimates **(d)** in patients with non-lymphoma malignancies who received Sintilimab (Arm B) and who received Pembrolizumab (Arm D).

Regarding the subset of 13 patients with advanced or recurrent lymphoma, median PFS and OS remained not reached for both Sintilimab-treated patients in Arm A and Pembrolizumab-treated patients in Arm C (**Figures 2a, b**). In the subset of 62 patients with advanced or recurrent non-lymphoma cancers, the median PFS was significantly longer at 15.0 months for Sintilimab-treated individuals in Arm B compared to 3.5 months for those in Arm D receiving Pembrolizumab (P=0.04), and the median OS was not reached versus 10.0 months for patients in Arm B and Arm D (P=0.01) (**Figures 2c, d**).

**Figure 3:**
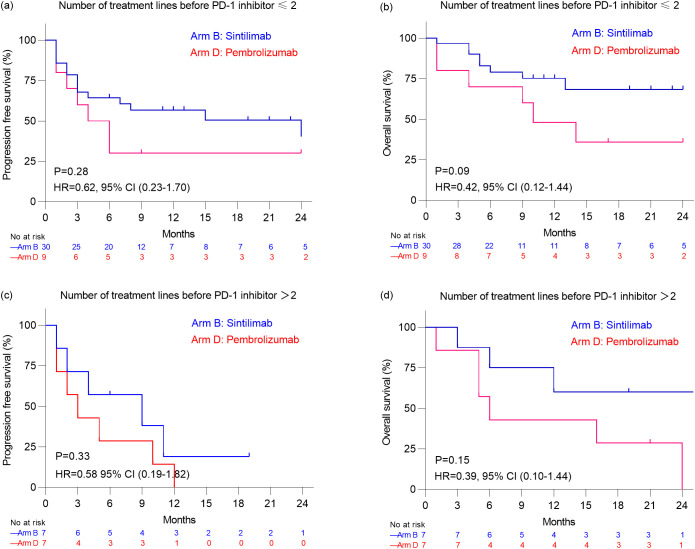
Kaplan–Meier analysis of progression free survival estimates **(a)** and overall survival estimates **(b)** in patients with non-lymphoma malignancies who received ≤2 treatment lines before PD-1 inhibitor. Kaplan–Meier analysis of progression free survival estimates **(c)** and overall survival estimates **(d)** in patients with non-lymphoma malignancies who received >2 treatment lines before PD-1 inhibitor.

Given 18.2%(8/44) patients in Arm B and 38.9%(7/18) patients in Arm D received>2 treatment lines before PD-1 inhibitor, subgroup analyses were also performed based on treatment line among patients with non-lymphoma group (**Figure 3**). However, among 39 patients who had received ≤ 2 prior treatment lines before PD-1 inhibitor therapy, there were no significant differences in PFS and OS between the Sintilimab group (n=30) and the Pembrolizumab group (n=9) (P=0.28 and P=0.09, respectively). Similarly, among 14 patients who had received > 2 prior treatment lines, there were no significant differences in PFS and OS between the Sintilimab group (n=7) and the Pembrolizumab group (n=7) (P=0.33 and P=0.15, respectively) (**Figure 3**).

## Discussion

Although some studies have demonstrated that PD-1 inhibitor-based regimens are potentially effective and tolerable in pediatric malignancies, it remains uncertain as to whether toxicity and efficacy are comparable between these PD-1 inhibitors in the real world (5, 8, 10–14). It is crucial to emphasize the significance of different PD-1 inhibitors in meeting the requirements of pediatric patients with advanced and recurrent malignancies. A recent phase 2, randomized, controlled trial demonstrated that Sintilimab exhibited similar efficacy and safety to Pembrolizumab in patients with advanced non-small cell lung cancer, regardless of PD-L1 expression levels (9). Additionally, a real-world study in China highlighted that Sintilimab was more commonly used than Pembrolizumab, with 46.2% of patients receiving Sintilimab and 7.5% receiving Pembrolizumab among 93 pediatric patients with malignancies (8). Our study addressed a critical knowledge gap by directly comparing Sintilimab and Pembrolizumab in a heterogeneous real-world cohort, where factors such as comorbidities, prior treatment burden, and irregular monitoring may influence outcomes. Our study revealed comparable safety and efficacy profiles for Sintilimab and Pembrolizumab in pediatric patients with malignancies.

Our study highlights a novel concern regarding cardiovascular toxicity in pediatric patients receiving PD-1 inhibitors. We observed relatively high rates of cardiovascular adverse events (CVAEs) following immune checkpoint inhibitor therapy, with incidence rates of 26% in the Sintilimab group and 40% in the Pembrolizumab group, primarily manifesting as arrhythmia. This finding underscores the complexity of immune-related adverse events in pediatric populations. PD-1 inhibitors enhance anti-tumor immune responses by activating T cells but may trigger excessive immune reactions leading to immune-related adverse events (15, 16). There are significant differences between children and adults in physiology, immune system development, drug metabolism and so on. As children’s immune systems are still developing, both the incidence and severity of immune-related adverse reactions may differ. Endocrine issues such as thyroid dysfunction and pituitary abnormalities could potentially impact growth and development (17). Unfortunately, there is very limited data on immune-related adverse events in pediatric patients receiving immune checkpoint inhibitor therapies (16). Previous clinical trials reported hypothyroidism incidence of 10%-20% and colitis incidence of 5%-10% in children treated with Pembrolizumab, with type 1 diabetes and myocarditis being rare but severe immune-related adverse events (3, 5–10, 12, 13, 18–22). It has been shown that Sintilimab-associated hypothyroidism occurred in 7% of cases (NCT04400851) (6). In our study, both Sintilimab and Pembrolizumab demonstrated higher rates of thyroid dysfunction compared to corresponding clinical trial data (**Supplementary Table 1**). However, previous clinical trials have not provided detailed reports on CVAEs following immunotherapy (3, 5–10, 12, 13, 18–22). Although immune-related cardiac adverse reactions are rare in children, they carry potentially fatal consequences. Clinicians should maintain high vigilance for non-specific symptoms such as arrhythmia and elevated cardiac enzymes.

Our study found that both sintilimab and pembrolizumab were generally well-tolerated, with hematological treatment-related adverse events (TRAEs) being the most common in both groups. The incidence of grade 3–4 hematologic TRAEs was similar between the two groups, and no severe infusion reactions or grade ≥3 non-hematologic TRAEs were observed. The incidence of non-hematologic AEs, such as fever, thyroid dysfunction, pneumonia, fatigue, increased ALT/AST, gastroenteritis, and rash showed no significant difference between Sintilimab group and Pembrolizumab group. The higher incidence of TRAEs in our study compared to clinical trials (eg, NCT04400851 for sintilimab and KEYNOTE-051 for pembrolizumab) may be attributed to the real-world setting, where patients often have more comorbidities, worse general conditions, and irregular disease monitoring (**Supplementary Table 1**) (5).

Among patients with lymphoma in our study, an ORR of 55.6% was achieved, including CR in 5 patients and PR in 3 patients, resulting in a DCR of 88.9%. These results are comparable to those of the phase I study of sintilimab in pediatric patients (NCT04400851), where the ORR and DCR were 60.0% and 100%, respectively (6). Patients treated with Pembrolizumab had an ORR and DCR of 75.0%, with 2 patients achieving CR and one achieving PR. These findings align with the phase I/II KEYNOTE-051 study (NCT02332668), showing an ORR of 60.0% among 15 patients with relapsed or refractory Hodgkin lymphoma (5). The median follow-up time for lymphoma patients in our study was 17 months, with median PFS and OS not reached for both Sintilimab and Pembrolizumab groups. The favorable prognosis of lymphoma patients indicated the need for further follow-up and long-term monitoring of thyroid and cardiac function. Our study contributes valuable real-world data on Sintilimab and Pembrolizumab in pediatric lymphoma, highlighting their potential efficacy in this population.

Among patients with non-lymphoma malignancies in our study, the ORR was 40.5% for Sintilimab and 25.0% for Pembrolizumab, while the DCR was 50.0% for Sintilimab and 35.0% for Pembrolizumab. There were no significant differences in ORR and DCR between the two groups. Although the median PFS and OS were significantly longer in the Sintilimab group (PFS: 15.0 months *vs*. 3.5 months, P = 0.04; OS: not reached *vs*. 10.0 months, P = 0.01), this difference may be related to the earlier initiation of PD-1 inhibitor therapy in the Sintilimab group, with a median of 1 treatment line before PD-1 inhibition compared to 2 in the Pembrolizumab group. When stratified by the number of treatment lines before PD-1 inhibition, no significant differences in PFS and OS were observed between the two groups among patients with ≤2 treatment lines or >2 treatment lines (all P values > 0.05). These findings suggest that the timing of PD-1 inhibitor administration may influence treatment outcomes. In the phase I trial of Sintilimab, an ORR of 21.4% and DCR of 35.7% were observed among 14 pediatric patients with non-lymphoma malignancies, with a median PFS of 1.8 months (6). Similarly, in the KEYNOTE-051 study, which evaluated Pembrolizumab in 136 pediatric patients with non-lymphoma malignancies, the ORR was 5.9%, the DCR was 26.5%, the median PFS was 1.9 months (5). In addition to these clinical trials, several studies have explored the efficacy of other PD-1 or PD-L1 inhibitors in pediatric patients with solid tumors. For instance, in the ESMART study (NCT2813135), which evaluated Nivolumab in children with relapsed/refractory solid tumors, a DCR of 20% was reported among 13 patients, with a median PFS of 1.7 months and OS of 3.4 months (21). The CHECKMATE 908 study (NCT03130959) reported a DCR of 30.8% among 85 pediatric patients with high-grade central nervous system malignancies treated with Nivolumab, with a median PFS of 1.7 months and OS of 11.7 months (12). In the iMATRIX study, which assessed Atezolizumab in pediatric malignancies, a DCR of 12.0% was observed among 75 patients with non-lymphoma malignancies (13). Furthermore, a DCR of 20.9% with Camrelizumab was reported in 43 pediatric patients with advanced osteosarcoma, with a median PFS and OS of 6.2 months (18). Collectively, these studies indicate that PD-1 or PD-L1 inhibitors generally exhibit limited efficacy in pediatric patients with non-lymphoma malignancies, which may be attributed to factors such as low tumor mutational burden or negative PD-L1 expression in the tumor microenvironment. Further optimization of treatment strategies is needed.

There are several limitations in this study. Firstly, the sample size was relatively small. Secondly, it was a retrospective study and thus had some information bias. In addition, the follow-up time was shorter in the Sintilimab group compared with the Pembrolizumab group with a median follow-up of 7 months versus 9.5 months. The study was also limited by the lack of racial diversity because it only included Asian pediatric patients.

In this real-world retrospective study, Sintilimab and Pembrolizumab both exhibited satisfactory overall safety profiles and anti-tumor activity. Notably, better anti-tumor activity was observed in patients with lymphoma compared to those with other malignancies. However, the modest efficacy observed in non-lymphoma malignancies underscores the need for further investigation into predictive biomarkers and combination therapies to enhance treatment outcomes. Long-term follow-up and enhanced monitoring of cardiac function, endocrine function and growth development are essential for pediatric patients receiving these treatments. Further prospective studies are needed to determine the optimal treatment strategies for pediatric patients with diverse malignancies.

## Data Availability

The raw data supporting the conclusions of this article will be made available by the authors, without undue reservation.
