# Supplementary material for: Safety and efficacy of sintilimab versus pembrolizumab in the treatment of advanced or recurrent pediatric malignancies: a real-world study in China
Source: Front Immunol. 2025 Jun 6;16:1608844. doi: 10.3389/fimmu.2025.1608844 (PMC12179053; doi:10.3389/fimmu.2025.1608844)
Supplement: Supplementary file 1 [file Table1.docx]

| Table 1. Summary of treatment-related adverse effects of this study, NCT04400851 and KEYNOTE-051 | | | | | | |
| --- | --- | --- | --- | --- | --- | --- |
| Adverse event | Sintilimab group(n=53) | NCT04400851  (Sintilimab, n=29) | P  value | Pembrolizumab group (n=20) | KEYNOTE-051  (Pembrolizumab, n=87) | P  value |
| Anemia* | 41(77.4%) | 10(34.5%) | ＜0.01 | 17(85.0%) | 12(13.8%) | ＜0.01 |
| Decreased white blood cell count* | 37(70.0%) | 4(13.8%) | ＜0.01 | 12(60.0%) | 6(6.9%) | ＜0.01 |
| Decreased neutrophils count* | 32(60.4) | 3(10.3%) | ＜0.01 | 12(60.0%) | 3(3.5%) | ＜0.01 |
| Decreased  platelets* | 26(49.1%) | 4(13.8%) | ＜0.01 | 10(50.0%) | 4(4.6%) | ＜0.01 |
| Fever | 18(33.9%) | 11(37.9%) | 0.72 | 2(10.0%) | 11(12.6%) | 1.00 |
| Fatigue | 13(24.5%) | 2(6.9%) | 0.05 | 7(35.0%) | 12(13.8%) | 0.06 |
| Anorexia* | 23(43.4%) | 2(6.9%) | ＜0.01 | 6(30.0%) | 4(4.6%) | ＜0.01 |
| Nausea | 4(7.6%) | 1(3.5%) | 0.64 | 4(20.0%) | 8(9.2%) | 0.32 |
| Thyroid dysfunction | 15(28.3%) | 4(14.0%) | 0.14 | 8(36.4%) | 14(16.1%) | 0.04 |
| Increased ALT/AST | 15(28.3%) | 6(20.1%) | 0.45 | 8(40.0%) | 16(18.4%) | 0.07 |
| Pneumonia* | 18(34.0%) | 1(3.5%) | ＜0.01 | 3(15.0%) | 2(2.3%) | 0.04 |
| Pleural effusion | 1(1.9%) | / | - | 1(5.0%) | 2(2.3%) | 0.47 |
| Gastroenteritis | 2(3.8%) | / | - | 3(15.0%) | 3(3.5%) | 0.08 |
| Abdomen pain | 6(11.3%) | 3(7.0%) | 1.00 | 2(10.0%) | 7(8.1%) | 1.00 |
| Rash | 2(3.0%) | 3(7.0%) | 0.34 | 1(5.0%) | 4(4.6%) | 1.00 |
| *Treatment-related adverse effects that P value＜0.01. ALT: Glutamic-pyruvic transaminase. AST: Glutamic oxalacetic transaminase. | | | | | | |
|  | | | | | | |
